# Minimizing Stress in White Sharks: Non-Invasive Epidermal Biopsies for Isotopic and Vitellogenin Analyses

**DOI:** 10.3390/biology14020192

**Published:** 2025-02-13

**Authors:** Guia Consales, Tommaso Campani, Agata Di Noi, Marco Garofalo, Eduardo Di Marcantonio, Francesca Romana Reinero, Silvia Casini, Luigi Dallai, Emilio Sperone, Letizia Marsili, Primo Micarelli

**Affiliations:** 1 Department of Physical Sciences Earth and Environment, University of Siena, 53100 Siena, Italy; guia.consales@unisi.it (G.C.); marco.garofalo@student.unisi.it (M.G.); silvia.casini@unisi.it (S.C.); letizia.marsili@unisi.it (L.M.); primo.micarelli@gmail.com (P.M.); 2 Santa Chiara Lab, University of Siena, 53100 Siena, Italy; agata.dinoi2@unisi.it; 3 Department of Earth Sciences, “Sapienza” University of Rome, 00185 Rome, Italy; eduardo.dimarcantonio@uniroma1.it (E.D.M.); luigi.dallai@uniroma1.it (L.D.); 4 Sharks Studies Center—Scientific Institute, 58024 Massa Marittima, Italy; ricerca@centrostudisquali.org; 5 Department of Biology, Ecology and Earth Sciences, University of Calabria, 87036 Rende, Italy; emilio.sperone@unical.it; 6 Centro Interuniversitario di Ricerca Sui Cetacei (CIRCE), University of Siena, 53100 Siena, Italy

**Keywords:** white shark, epidermal biopsy, vitellogenin, stable isotopes, endocrine disruption, conservation, trophic ecology

## Abstract

The great white shark (*Carcharodon carcharias*), a keystone predator vital to marine ecosystem stability, is increasingly exposed to anthropogenic threats, including endocrine-disrupting chemicals (EDCs). This study highlights the use of non-invasive epidermal biopsies to assess physiological and ecological parameters in 28 live specimens sampled from the Dyer Island Nature Reserve, South Africa. Epidermal tissue was analyzed for vitellogenin (Vtg), a biomarker of estrogenic exposure, while dermal tissue was used for stable isotope analyses of carbon and nitrogen, essential for understanding the feeding habitat of white sharks. Vitellogenin, typically restricted to sexually mature females, was unexpectedly detected in males and immature females, indicating significant exposure to estrogenic pollutants. This finding raises concerns about the potential reproductive and population-level impacts on this vulnerable species. Stable isotope analyses confirmed that dermal tissue alone is sufficient for trophic studies, eliminating the need for deeper muscle sampling. By demonstrating that epidermal and dermal tissues provide critical data for both biomarkers and isotopic studies, this research supports the adoption of minimally invasive techniques. Shallower biopsies reduce stress on the animals, making this method a valuable tool for conservation research and management of *C. carcharias*.

## Simple Summary

White sharks (*Carcharodon carcharias*), apex predators crucial to marine ecosystems, face numerous anthropogenic threats, including endocrine-disrupting chemicals, primarily acquired through their diet. To advance their conservation, we investigated the use of minimally invasive epidermal biopsies for isotopic and vitellogenin analyses. These techniques provide critical insights into shark feeding habits and pollutant exposure without significantly stressing the animals. Our findings suggest widespread estrogenic exposure in South Africa’s white shark population, raising concerns about reproductive and ecological impacts.

## 1. Introduction

White sharks (*Carcharodon carcharias*), as apex predators, play a pivotal role in regulating marine ecosystems by controlling prey populations and facilitating nutrient cycling. However, they face significant threats from overfishing, habitat destruction, and exposure to anthropogenic pollutants, including endocrine-disrupting chemicals (EDCs) such as some polychlorinated biphenyls (PCBs) and other organochlorine pesticides [1,2,3,4]. EDCs can inhibit hormonal pathways, leading to reproductive impairments and population declines in affected species [5,6,7,8,9].

A biomarker is defined as “a biochemical, cellular, physiological, or behavioural changes that can be measured in a tissue, biological fluid, or whole organisms that provide evidence of exposure to, and/or toxic effects of, one or more contaminants” [10]. To evaluate the impact of pollutants on sharks, biomarkers are widely used [1,2,11,12,13]. Among the most effective biomarkers for endocrine disruption is vitellogenin (Vtg), a phospholipoglycoprotein precursor of egg yolk [14,15]. Vtg is synthesized in the liver under the influence of estrogens and is normally found only in sexually mature females [16]. However, when specimens are exposed to estrogen-mimicking pollutants, such as organochlorines or plasticizers, abnormal Vtg production can occur in males and immature specimens [14,15]. This makes Vtg a sensitive and reliable marker for estrogenic exposure and endocrine disruption. Elevated Vtg levels in immature and male white sharks have been associated with exposure to estrogenic pollutants, raising concerns about the long-term reproductive impacts, including skewed sex ratio and reduced fertility, which could threaten population stability [1]. While Vtg levels are commonly assessed through invasive plasma sampling in vertebrates [17,18,19] and non-invasive mucus sampling in teleost fish [20], the unique physiological characteristics of elasmobranchs preclude the use of mucus as a non-invasive sampling matrix. Consequently, alternative tissues must be explored. Skin biopsies, a well-established method for evaluating cytochrome CYP1A and 1B activity, offer a promising avenue for Vtg analysis in elasmobranchs. The ability to detect and measure such disruptions through biomarkers like Vtg is critical for understanding and mitigating the impact of chemical pollution on vulnerable marine species and thus provides important insights for conservation measures.

Stable isotope analysis (SIA) was predominantly used in earth sciences but has become widely applied in ecological studies over the past two decades [21,22]. Biologists now employ SIA in paleoecology, ecosystem studies, physiological ecology, and population dynamics [23,24,25].

Studying how stable isotopes fractionate into animal tissues provides insights into the tracking of dietary changes over time [26] and the understanding of the use of assimilated resources on various timescales, depending on the tissue’s metabolic turnover rate [27]. Isotopes of carbon (δ^13^C) and nitrogen (δ^15^N) are commonly analyzed in trophic studies. The δ^13^C values reveal information about habitat use and dietary sources, distinguishing inshore from pelagic feeding environments [28]. In contrast, δ^15^N values indicate the trophic position of the organism, reflecting its role in the food chain [29].

Animal tissues like plasma, muscle, bone, and skin are typically used for isotopic studies. Tissues with rapid turnover rates, such as plasma, provide short-term dietary information, while slower turnover tissues like dermis and bone reflect long-term dietary habits [30]. Dermal tissue, therefore, holds significant potential as a valuable matrix for studying long-term feeding ecology. In white sharks, SIA has been applied to vertebrae to investigate ontogenetic dietary changes [31,32,33,34]. However, dermal tissue may also represent a viable alternative. While the turnover rate of the dermis in white sharks remains unknown, it is presumed to be slow, making it a reliable source of dietary information over extended periods [35]. Traditionally, muscle tissues are used for SIA to infer trophic position and habitat use [36,37]. However, such sampling methods are more invasive and may harm or stress these vulnerable species. This study aims to validate epidermal biopsies (1 cm; [38]) as a minimally invasive alternative for assessing pollutant exposure and dietary habits, parameters closely associated, as the intake of contaminants is directly correlated with the type of diet consumed, offering an effective alternative method for white shark conservation.

## 2. Materials and Methods

### 2.1. Study Area

**Figure 1:**
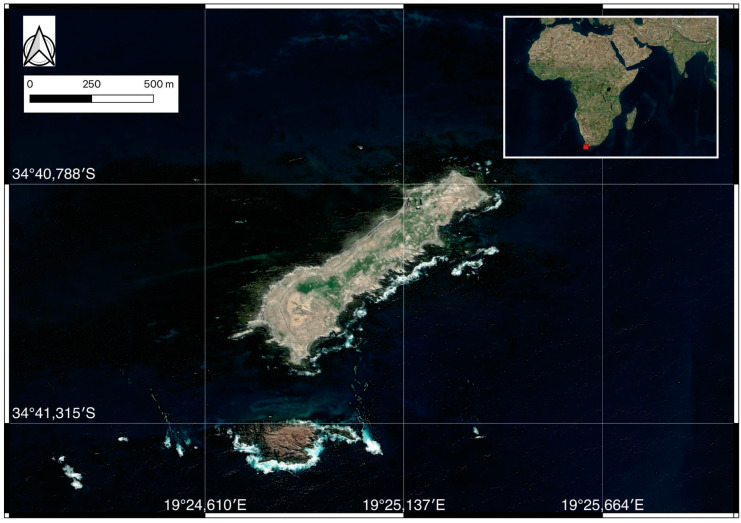
Nature Reserve of Dyer Island where samplings occurred highlighted with the red dot in the upper-right figure. The map has been created through Q-GIS LTR 3.22 software.

Fieldwork was conducted in Dyer Island Nature Reserve, South Africa, a global hotspot for white sharks known for its high predator–prey interaction rates [39]. The Nature Reserve of Dyer Island is located 7.5 km off the coast of Gansbaai (34°41′ S; 19°24′ E), South Africa. This Reserve includes Dyer Island and Geyser Rock (Figure 1): the first is a low-profile island ca. 1.5 km long and 0.5 km wide and is important and characterized by the presence of different seabird colonies; in contrast, the second is ca. 0.5 km long and 180 m wide, and it hosts a colony of Cape fur seals *Arctocephalus pusillus pusillus* (Schreber 1775). The Nature reserve is located in an area called Agulhas Bioregion, and it is the meeting point between the Benguela Current, which is the eastern boundary current of the subtropical vortex located in the South Atlantic Ocean, and the Agulhas Current, which is the current forming the western limit of the Indian Ocean. During the summer season, intensified southeasterly trade winds result in upwelling, causing cold waters of Benguela origin to enter the bay [40]. The upwelling occurring along the coast results in high biological productivity, which in turn supports large fish stocks, including pilchard, anchovy, and hake [41].

### 2.2. Sex Determination and Sample Collection

Observations and sample collection occurred between March and July 2015 aboard the Slashfin, a 14 m-long vessel operated by Marine Dynamics. Sex determination was performed on-site during the collection of skin biopsies. The identification was conducted on board through cage-diving observations of the pelvic fin area. Males were identified by the presence of claspers, while females were confirmed by the absence of these structures. Sexual maturity was determined based on body size, with males considered sexually mature at lengths exceeding 3.5 m and females at lengths exceeding 4.5 m [42]. Less invasive epidermal biopsies were obtained from 28 sharks (9 males, 15 females, and 4 unidentified) using a biopsy pole with a modified tip to penetrate the skin layer of each white shark, with great care taken to sterilize the tip by flaming. The pole allowed precise tissue collection from free-swimming sharks attracted near the vessel using chum (fish offal and oils). Biopsies were immediately preserved in liquid nitrogen and later stored at −80 °C until analysis.

### 2.3. Electrophoresis and Immunoblotting for Vitellogenin Quantification with Stain-Free Technology

Biopsy samples were shredded using sterile scalpels and transferred into 2 mL tubes. Wilson buffer (25 mM HEPES, 1 mM EDTA, 5 mM EGTA, 0.02% NaN_3_, 20 mM Na_2_MoO_4_, 1 mM DTT, 10 μL/mL protease inhibitors, and 10% glycerol) 1:10 was added to the tubes, and samples were solubilized using a TissueLyser (Qiagen, Hilden, Germany) twice: first for 4 min at 20 Hz and then for 3 min at 20 Hz. The samples were centrifuged at 750× *g* for 10 min at 4 °C, followed by another centrifugation at 10,000× *g* for 20 min at 4 °C. The supernatant was collected, and protein concentration was determined using the BCA kit (Thermo Fisher Scientific, Waltham, MA, USA) spectrophotometrically at 562 nm. A series of BSA standards (0, 5, 10, 15, 20, 30, 40, and 50 μg) was prepared. Polyacrylamide gels were prepared using the “TGX Stain-Free™ FastCast™ Acrylamide Kit”. Unstained protein standards (Bio-Rad, Hercules, CA, USA) were used as molecular weight reference. Electrophoresis was run at 200 V using 1X TGS running buffer (Tris/Glycine/SDS, Bio-Rad). Finally, the gel was placed into the ChemiDoc MP Imaging System (Bio-Rad) and subjected to the Stain-Free Gel procedure (590/110 UV Trans) with auto-optimal exposure for 45 s. For immunoblotting, the separated proteins were transferred onto a nitrocellulose membrane using the “Trans-Blot Turbo” electrotransfer system (BioRad) at 1.3 A and 25 V, for 7 min. The membranes were checked in the ChemiDoc system to confirm proper transfer. After the transfer, the membranes were immersed in a blocking solution containing 5% Blotting-Grade Blocker (Bio-Rad) and 0.1% Tween 20 (Bio-Rad) in Tris-Buffered Saline (TBS). They were agitated at room temperature for one hour. The membranes were washed twice in 1X TBS with agitation for 5 min each. After removing the 1X TBS, the membranes were incubated with dilution buffer (20 mL 1X TBS, 20 μL Tween 20) and the primary polyclonal antibody “rabbit anti-sea bream vitellogenin” (BioSense Laboratories SA, Bergen, Norway) at a 1:1000 dilution for one hour with agitation. After two more washes with 1X TBS (5 min each), the secondary antibody “goat anti-rabbit IgG StarBright Blue 700” (BioRad) diluted in the dilution buffer was added. The membranes were agitated for one hour. Finally, the membranes were washed twice with 1X TBS (5 min each) and analyzed using the ChemiDoc (BioRad) system. Images of the blotting with the secondary antibody and stain-free blot (total proteins) were acquired. The images were analyzed using the Image Lab 6.1 software (BioRad). The stain-free blot images and the antibody-only blot images were compared. Lanes were detected and adjusted for each sample, and bands corresponding to antibody exposure were selected. At the end of the image processing, the normalized volume of each band was obtained relative to the total protein content of the sample.

### 2.4. Stable Isotope Analysis Methodology

After being freeze-dried, the samples were ground into a fine powder, and 0.3 mg of material was weighed into tin capsules. Isotopic analyses were performed using a Flash HT/CHN Elemental Analyzer (Thermo-Fisher Scientific) connected to a Delta V Advantage IRMS (Thermo-Fisher Scientific) operating in continuous flow mode [43,44]. The isotopic values were calibrated against laboratory standards and international standards IAEA-CH-6 and IAEA-CH-7. The results are reported in delta notation (‰) relative to the international standard V-PDB for carbon and Air N_2_ for nitrogen. Each sample has been replicated at least twice, and the standard error of the mean is generally below 0.20‰ both for carbon and nitrogen. Stable isotope analyses performed to constrain dietary shift in shark populations have employed different animal tissues, such as liver, muscle, and cartilage in the blue shark [36], anal and dorsal fin tissue in reef sharks [37], dermis and muscle in white sharks [35,45,46], and muscles and the liver in hammerhead sharks [47]. Consistent although scattered δ^13^C and δ^15^N data have been reported in the literature, likely resulting from diet variations and migratory behavior. The stable isotope values of multiple tissues show systematic isotope differences and likely indicate variations in the turnover of stable isotopes in distinct body tissues. In this paper, epidermal tissues were measured for carbon and nitrogen isotope composition to verify whether these minimally invasive samples may be consistent in isotopic composition with other body tissues.

### 2.5. Data Analysis

Statistical relationships between Vtg and length have been investigated through Spearman’s r correlation test in GraphPad Prism 8.0.2., while δ^15^N, δ^13^C, length, and gender have been investigated through the General Linear Model in the R 4.2.2 software environment.

## 3. Results

Of the 28 collected samples, 27 were analyzed for stable isotopes, while only 18 were analyzed for vitellogenin (Vtg) due to insufficient material in some samples to ensure accurate protein quantification. One biopsy lacked adequate tissue for any analysis. The sex and length data as well as results from SIA and Vtg quantification of the sampled specimens are summarized in Table 1.

Of the 27 sampled specimens, 15 were identified as females, eight as males, and four as “not determined” specimens for whom sex could not be established.

On average, the sampled females measured 3.6 ± 0.6 m in length, the males 3.3 ± 0.4 m, and the “not determined” specimens had an average length of 3.0 ± 0.3 m. Among all sampled specimens, only three (WS12-M, WS15-M, and WS8-F) were identified as sexually mature.

### 3.1. Vitellogenin Quantification

**Figure 2:**
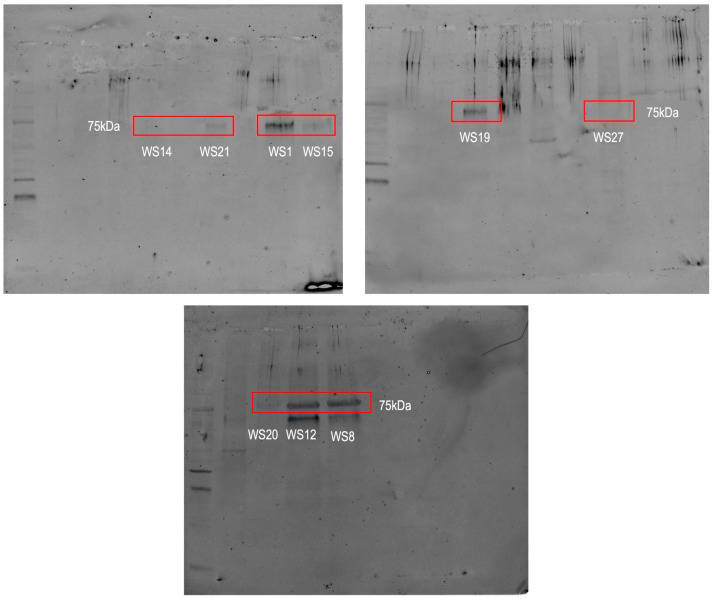
Western blot analysis for vitellogenin (Vtg) detection in white shark (*Carcharodon carcharias*) epidermal tissue samples. Three separate blots are shown, representing different sets of samples (WS14, WS21, WS15, WS1, WS19, WS27, WS20, WS12, and WS8). The bands corresponding to Vtg (~75 kDa) are highlighted with red boxes. Original images (Figures S1–S3) are available in Supplementary Material.

Only nine out of 18 specimens exhibited measurable vitellogenin (Vtg) expression (Figure 2). Among them, five females exhibit high levels of this protein, but only one of these specimens, identified as “WS8”, is sexually mature. The notable finding is the presence of this protein in four male specimens: “WS12”, “WS15”, “WS20”, and “WS21”. The results indicate that the highest Vtg level among males was found in the individual “WS12”, while the highest level among females was observed in “WS19”.

**Figure 3:**
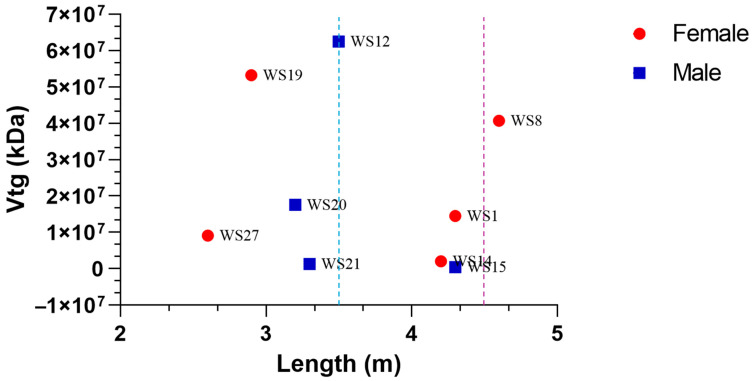
Vitellogenin and length relationship in male and female specimens. Dashed lines represent sexual maturity for males (3.5 m, blue) and females (4.5 m, purple).

The Vtg values were then plotted against the body length for both males and females, highlighting the body lengths at which sexual maturity is estimated with two dashed lines (blue for males and red for females) (Figure 3). No significant statistical correlation was found between Vtg concentrations and body lengths, neither for males nor for females. What stands out is that both immature and mature males, as well as immature females, have Vtg levels comparable to or even higher than the only female (WS8) considered sexually mature based on her body size.

### 3.2. Stable Isotope Analysis

**Figure 4:**
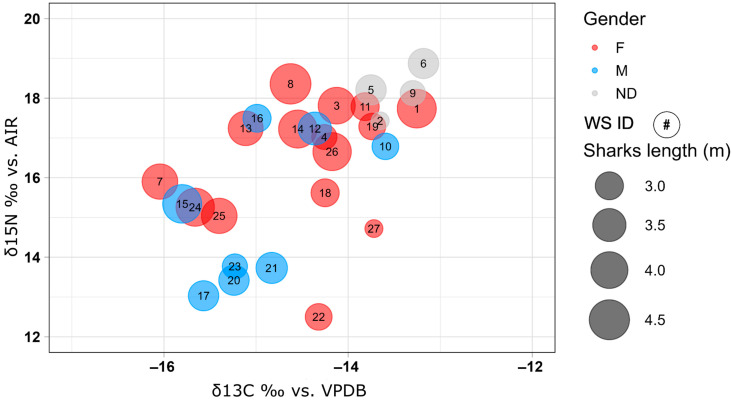
Isotopic results for δ^15^N and δ^13^C are plotted in this graph. The size of the dots represents the length of the sharks, and the number inside the dot is the shark sample ID.

**Figure 5:**
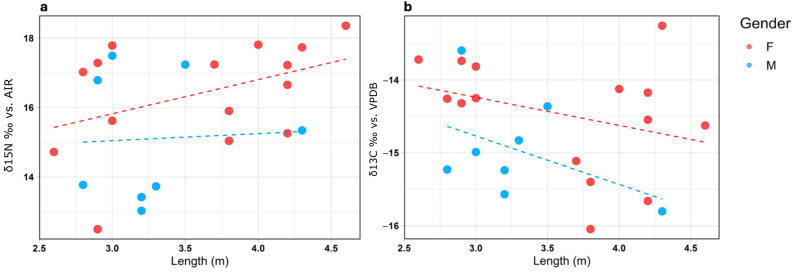
(**a**) Graph showing the relationship between nitrogen isotopic ratios (δ^15^N ‰) and body length in female (red) and male (light blue); (**b**) Graph showing the relationship between carbon isotopic ratios (δ^13^C ‰) and body length in female (red) and male (light blue).

The mean isotopic value from dermal tissue was −14.46 ± 0.87‰ for carbon (δ^13^C) and 16.28 ± 1.83‰ for nitrogen (δ^15^N). The analyses revealed a significant relationship between δ^15^N and δ^13^C in the analyzed shark samples, indicating a positive correlation between the two isotopic variables with a *p*-value = 0.002 and R2 for the regression line of 0.315 (Figure 4). No significant relationships were observed between δ^15^N and the total body length, nor were there interactions with sex (*p* > 0.05). Conversely, for δ^13^C, a significant relationship with individual length was identified (*p* = 0.031, R2 = 0.1). Despite this association, no significant interaction between length and sex was found for any of the isotopic variables analyzed (Figure 5). To interpret these data, specimens were clustered based on sex and size into six categories: males > 3 m, males < 3 m, females > 3 m, females < 3 m, undetermined > 3 m, and undetermined < 3 m (Table 2). The threshold of 3 m was chosen because ontogenetic changes, including dietary shifts, occur at approximately this size. Around this length, white sharks’ teeth develop broader bases and serrated edges, enabling them to transition from a diet primarily consisting of fish and small prey to one dominated by marine mammals and cetaceans [48].

## 4. Discussion

This study provides new insights into the physiological and ecological status of white sharks in South Africa, highlighting the importance of adopting minimally invasive techniques for biomarker and isotopic analyses. One of the most notable and interesting results of this study was the detection of vitellogenin (Vtg) in male white sharks and immature females. Being a protein typically produced in the liver of sexually mature females under estrogen stimulation, its presence in males and immature females strongly suggests exposure to estrogenic substances, such as endocrine-disrupting chemicals (EDCs) [49]. This result is consistent with earlier findings by Marsili et al. [1], who, in the same population of this study, identified POPs with endocrine-disrupting properties and documented Vtg expression in a male individual. The abnormal detection of Vtg in South African white sharks is a clear biomarker of estrogenic exposure [49] and suggests that exposure to such substances could lead to significant physiological effects damaging reproduction and potentially compromise the conservation of the species [50]. This finding highlights the importance of evaluating the effects of environmental contaminants with endocrine-disrupting potential through non-invasive biomarkers, such as Vtg in dermal tissue, to accurately assess the conservation status of at-risk species like the white shark.

The results of the isotopic analyses on dermal tissue showed that this methodology, in addition to being minimally invasive, provides isotopic values consistent with data obtained from the same specimens’ muscle tissues [46]. The linear regression model applied to the two analyzed isotopes (nitrogen and carbon) revealed a good correlation (*p* = 0.002, R2 = 0.315) (Figure 4). Furthermore, the mean isotopic values of organisms clustered into <3 m and >3 m categories align with the conventional expectation of higher δ^13^C and, more specifically, δ^15^N values for mature specimens [33,51]. This trend supports the idea that isotopic shifts in larger specimens reflect changes in diet or habitat associated with ontogenetic development.

Despite these promising results, going deeper into the analyses, the weak correlation between length and stable isotopes (*p* > 0.05, R2 = 0.1 for δ^15^N, all organisms versus length) complicates the ecological interpretation of the dataset. While this information may have limited ecological significance, it corroborates similar results obtained from the same organisms but sampled for muscle tissues [46], leading to a new insight into the reliability of dermal tissue for stable isotope analysis. Specifically, the correlation between isotopic values and length for males and females appears comparable (Figure 5). Moreover, a graphically increasing trend for δ^15^N from females (Figure 5a), although it is not statistically appreciable, is evident, as it was for French et al. [46] (*p* = 0.24, R2 = 0.03 for δ^15^N in females vs. length; *p* = 0.31, R2 = 0.05 for δ^15^N in males vs. length; *p* = 0.031, R2 = 0.1 for δ^13^C in females vs. length; *p* = 0.041, R2 = 0.12 for δ^13^C in males vs. length).

The absence of significant isotopic differences across sex and size categories suggests similar feeding habits among specimens in this white shark population, contrary to the established literature on dietary shifts in white sharks [37,48,52,53,54]. As previously demonstrated, dietary changes are either in progress or have been completed by 3 m size in transient Gansbaai populations [55]. Despite the known ontogenetic dietary changes in white sharks, where larger specimens typically shift to marine mammals, this study found no evidence of size-based dietary variation within the sampled population. Our findings align with the study by French et al. [46], who reported similar trends in isotopic analyses conducted on muscle tissues. Only samples from females below and above 3 m in size show variations in nitrogen isotope composition (14.78 ± 1.80‰ and 16.87 ± 1.07‰, respectively), whereas no significant differences are observed in carbon isotope composition, either based on sex or size. A similar difference has been reported by Graham et al. [56] for coastal and open-ocean foraging zones in the Northwest Atlantic, possibly reflecting different ocean feeding environments. Dermal tissue, being metabolically less active than tissues like the liver or blood, has a slower isotope incorporation rate, which may depend on body mass. This could explain the observed differences in nitrogen isotopes in larger female sharks (>3 m). The consistency in isotopic results between dermal and muscle tissues reinforces the reliability of dermal tissue as a minimally invasive alternative for ecological studies.

## 5. Conclusions

This study demonstrates the feasibility and importance of using minimally invasive techniques, such as epidermal biopsies, to collect critical ecological and physiological data from white sharks. The detection of vitellogenin (Vtg) in males and immature females highlights the persistent impact of endocrine-disrupting pollutants in marine environments. Elevated Vtg levels in these groups serve as a biomarker of estrogenic contamination, raising concerns about the long-term reproductive and ecological stability of the population. Since isotopic analyses suggest no dietary changes in the specimens investigated, it is assumed that the contamination input is currently relatively consistent and that it is responsible, or at least partially responsible, for the endocrine disruption highlighted by the Vtg levels. Overall, this study highlights the potential for dermal tissue to replace muscle tissue in isotopic research, while also raising interesting questions about the ecological dynamics of South African white sharks. This finding emphasizes the potential of dermal biopsies to replace more invasive sampling methods, reducing stress on these vulnerable species. Further investigation is essential to determine whether these observed trends are population-specific anomalies or part of broader behavioral adaptations. Lastly, the integration of vitellogenin and isotopic analyses in this study emphasizes the importance of combining biochemical and ecological approaches to evaluate the health and conservation status of vulnerable apex predators like white sharks.

## Figures and Tables

**Table 1:** Sample ID, biological parameters (sex and length), SI values, and Vtg expression of biopsied white shark specimens.

Sample ID	Sex	Length (m)	δ^15^N ‰	δ^13^C ‰	Vitellogenin (kDa)
WS1	F	4.3	17.74	−13.26	14,520,649
WS2	ND	2.6	18.87	−13.18	-
WS3	F	4	16.79	−13.60	0
WS4	F	2.8	17.79	−13.82	0
WS5	ND	3.2	15.90	−16.04	-
WS6	ND	3.2	18.36	−14.63	-
WS7	F	3.8	17.24	−14.36	0
WS8	F	4.6	17.24	−15.11	40,673,677
WS9	ND	2.8	18.13	−13.30	0
WS10	M	2.9	13.77	−15.23	0
WS11	F	3	17.22	−14.55	-
WS12	M	3.5	15.26	−15.66	62,489,877
WS13	F	3.7	15.34	−15.80	-
WS14	F	4.2	17.49	−14.99	2,019,391
WS15	M	4.3	15.04	−15.40	407,678
WS16	M	3	16.66	−14.17	-
WS17	M	3.2	14.72	−13.72	0
WS18	F	3	13.03	−15.57	-
WS19	F	2.9	15.62	−14.25	-
WS20	M	3.2	17.81	−14.12	17,551,193
WS21	M	3.3	17.02	−14.26	1,312,209
WS22	F	2.9	17.29	−13.74	0
WS23	M	2.8	18.21	−13.75	0
WS24	F	4.2	17.43	−13.65	0
WS25	F	3.8	13.42	−15.24	-
WS26	F	4.2	13.73	−14.83	-
WS27	F	2.6	12.50	−14.32	9,131,169

**Table 2:** Isotopic values (δ^15^N and δ^13^C, mean ± standard deviation) for white sharks grouped by sex (males, females, and undetermined) and size categories (<3 m and >3 m).

Sex	Size Category (m)	δ^15^N (‰)	δ^13^C (‰)
Undetermined	<3 >3	17.78 ± 0.35 18.54 ± 0.33	−13.47 ± 0.18 −13.47 ± 0.28
Males	<3 >3	15.28 ± 1.51 15.39 ± 1.72	−14.41 ± 0.82 −14.85 ± 0.65
Females	<3 >3	14.78 ± 1.80 16.87 ± 1.07	−14.35 ± 0.55 −14.66 ± 0.85

## Data Availability

The data presented in this study are available on request from the corresponding author.

## Supplementary Materials

The following supporting information can be downloaded at: https://www.mdpi.com/article/10.3390/biology14020192/s1.
