# Supplementary figures and images for: Minimizing Stress in White Sharks: Non-Invasive Epidermal Biopsies for Isotopic and Vitellogenin Analyses
Source: Biology (Basel). 2025 Feb 13;14(2):192. doi: 10.3390/biology14020192 (PMC11852308; doi:10.3390/biology14020192)

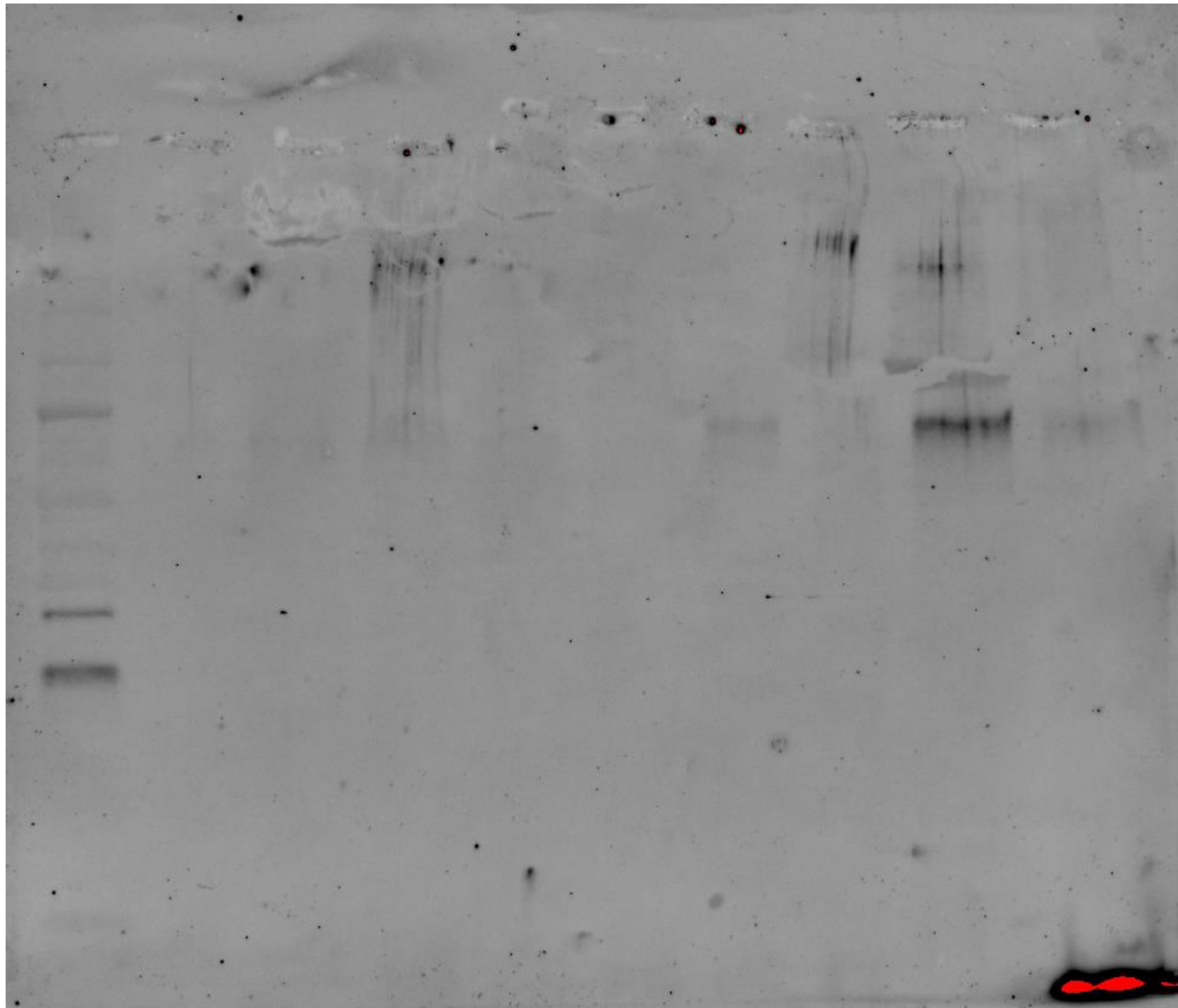

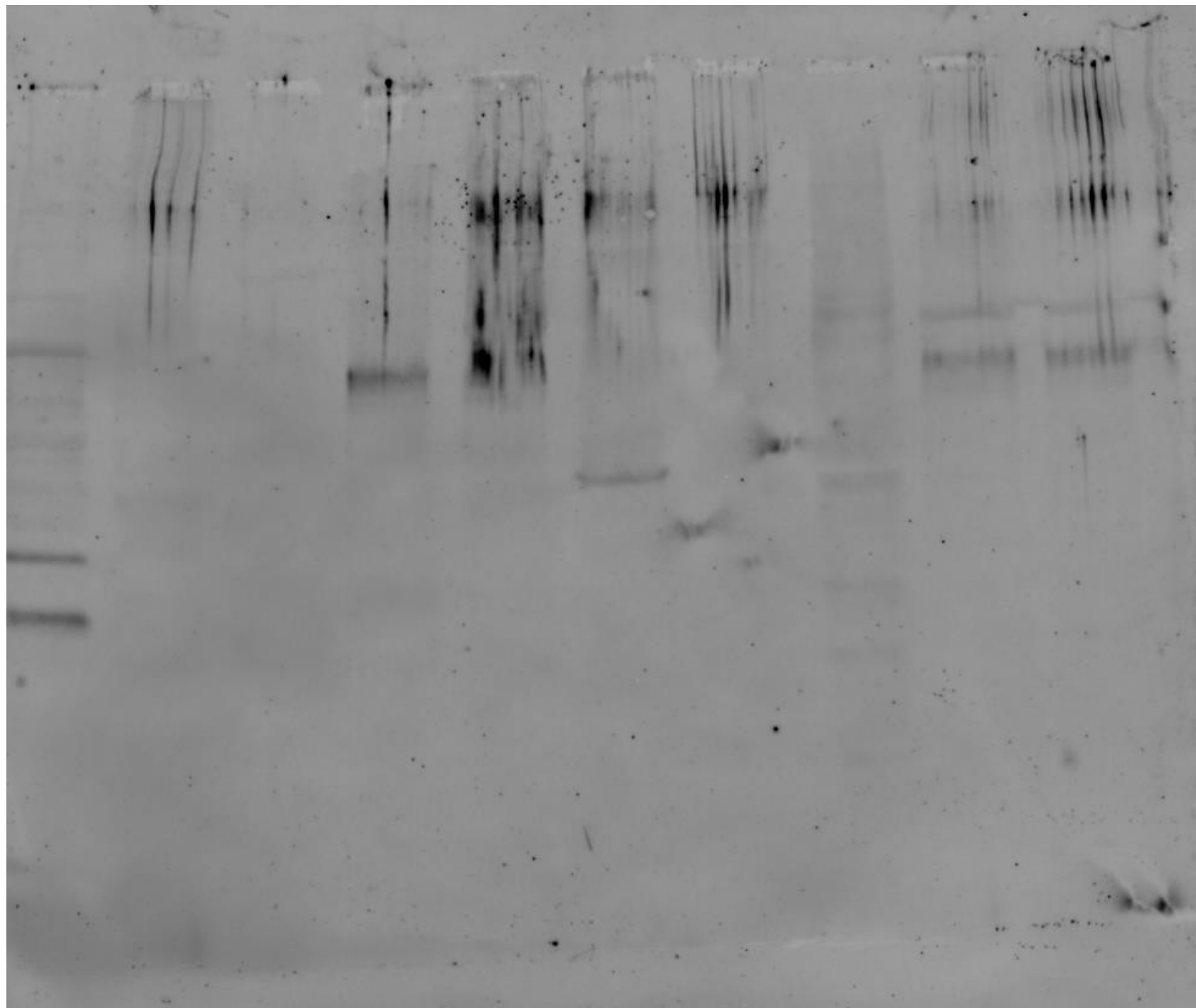

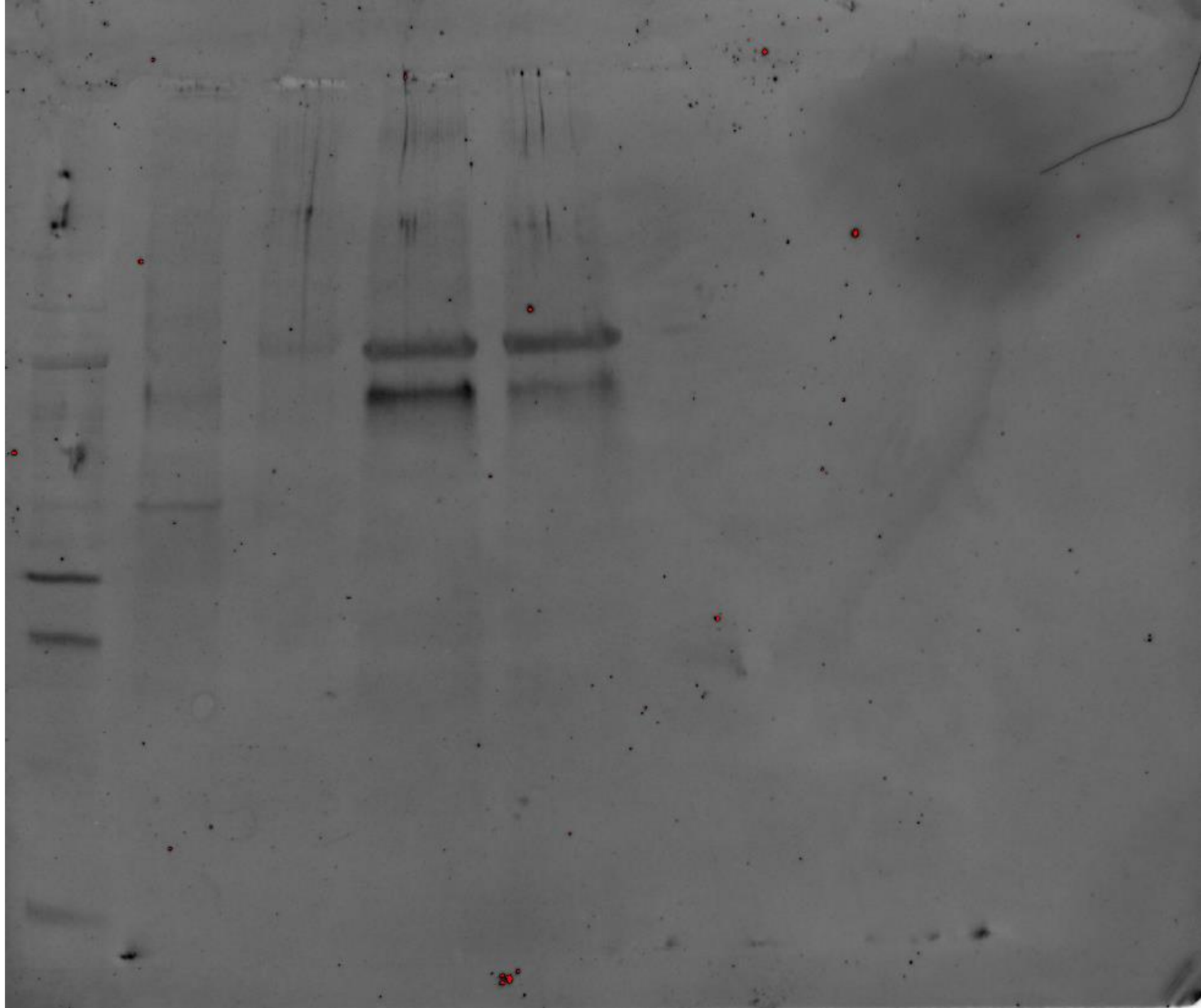

Supplement: Supplementary file 1 [file biology-14-00192-s001.zip › biology-3431610-supplementary.pdf]
